# Supplementary material for: Identifying amyloid-related diseases by mapping mutations in low-complexity protein domains to pathologies
Source: Nat Struct Mol Biol. 2022 May 30;29(6):529–36. doi: 10.1038/s41594-022-00774-y (PMC9205782; doi:10.1038/s41594-022-00774-y)
Supplement: Supplementary file 1 — Reporting Summary [file 41594_2022_774_MOESM1_ESM.pdf]

nature portfolio

Corresponding author(s): David S. Eisenberg

Last updated by author(s): March 1, 2022

Reporting Summary

Nature Portfolio wishes to improve the reproducibility of the work that we publish. This form provides structure for consistency and transparency in reporting. For further information on Nature Portfolio policies, see our Editorial Policies and the Editorial Policy Checklist.

Statistics

For all statistical analyses, confirm that the following items are present in the figure legend, table legend, main text, or Methods section.

n/a	Confirmed	
☐	☒	The exact sample size (*n*) for each experimental group/condition, given as a discrete number and unit of measurement
☐	☒	A statement on whether measurements were taken from distinct samples or whether the same sample was measured repeatedly
☒	☐	The statistical test(s) used AND whether they are one- or two-sided<br>*Only common tests should be described solely by name; describe more complex techniques in the Methods section.*
☐	☒	A description of all covariates tested
☒	☐	A description of any assumptions or corrections, such as tests of normality and adjustment for multiple comparisons
☐	☒	A full description of the statistical parameters including central tendency (e.g. means) or other basic estimates (e.g. regression coefficient) AND variation (e.g. standard deviation) or associated estimates of uncertainty (e.g. confidence intervals)
☒	☐	For null hypothesis testing, the test statistic (e.g. F, t, r) with confidence intervals, effect sizes, degrees of freedom and P value noted<br>*Give P values as exact values whenever suitable.*
☒	☐	For Bayesian analysis, information on the choice of priors and Markov chain Monte Carlo settings
☒	☐	For hierarchical and complex designs, identification of the appropriate level for tests and full reporting of outcomes
☒	☐	Estimates of effect sizes (e.g. Cohen's *d*, Pearson's *r*), indicating how they were calculated

Our web collection on statistics for biologists contains articles on many of the points above.

Software and code

Policy information about availability of computer code

Data collection	Zipper and LARKS score calculations were performed using publicly available code found on ZipperDB and LARKSdb, as detailed in the Data Availability section
Data analysis	GraphPad Prism (version 9.1.2) and Microsoft Excel for Mac (version 16.58) were used for all data analysis and statistics. Crystal structures were presented using UCSF Chimera (version 1.13.1). Atomic models were built in COOT (version 0.8.9.1). Model refinement was performed with phenix.real_space_refine (version 1.13-2998).

For manuscripts utilizing custom algorithms or software that are central to the research but not yet described in published literature, software must be made available to editors and reviewers. We strongly encourage code deposition in a community repository (e.g. GitHub). See the Nature Portfolio guidelines for submitting code & software for further information.

Data

Policy information about availability of data

All manuscripts must include a data availability statement. This statement should provide the following information, where applicable:

- Accession codes, unique identifiers, or web links for publicly available datasets
- A description of any restrictions on data availability
- For clinical datasets or third party data, please ensure that the statement adheres to our policy

The datasets generated and/or analyzed during the current study are provided as Source Data. Any additional data are available from the corresponding author.

All structural data have been deposited into the Worldwide Protein Data Bank (wwPDB) with the following accession codes: SGMGGIT (PDB 7K3C), SGMGCIT (PDB 7K3X), and GGYAGAS (PDB 7K3Y).

Calculations of zipper and LARKS propensity can be performed using our online web servers, which can be found at https://services.mbi.ucla.edu/zipperdb/ and https://srv.mbi.ucla.edu/LARKSdb/

Field-specific reporting

Please select the one below that is the best fit for your research. If you are not sure, read the appropriate sections before making your selection.

☒ Life sciences ☐ Behavioural & social sciences ☐ Ecological, evolutionary & environmental sciences

For a reference copy of the document with all sections, see nature.com/documents/nr-reporting-summary-flat.pdf

Life sciences study design

All studies must disclose on these points even when the disclosure is negative.

Sample size	All ThT assays were performed with three experimental replicates, and are generally considered sufficient for plate-reader based experiments to determine a standard deviation. This sample size has also been used in previous studies by our group (Cao et al. Cryo-EM structure and inhibitor design of human IAPP (amylin) fibrils. Nat. Struct. Mol. Biol (2020); Cryo-EM structure of hIAPP fibrils seeded by patient-extracted fibrils reveal new polymorphs and conserved fibril cores) as well as other groups (Wördehoff et al. α-Synuclein Aggregation Monitored by Thioflavin T Fluorescence Assay. Bio Protoc. (2018)).
Data exclusions	For the Zipper Score calculations in Fig. 1, all sequences containing prolines were excluded from data analysis. ZipperDB is unable to calculate an energetic score for amyloid structures containing prolines due to their disruptive effects on beta-sheet stacking
Replication	All attempts at replication were successful. For ThT assays and Western blots, experiments were performed with three independent experimental replicates. For EM images, at least 3 images were taken per condition
Randomization	No randomization was necessary for this study because investigators were comparing quantitative data under well controlled conditions. No human or animal subjects were used. For all ThT assays, samples were loaded to a single 96-well plate, treated equally, and the results were recorded using an automatic plate reader, leading to objective results that do not require randomization.
Blinding	Investigators were not blinded because conditions were well controlled and quantitatively measured/calculated in an unbiased manner, and not dependent on subjective interpretation or analysis.

Reporting for specific materials, systems and methods

We require information from authors about some types of materials, experimental systems and methods used in many studies. Here, indicate whether each material, system or method listed is relevant to your study. If you are not sure if a list item applies to your research, read the appropriate section before selecting a response.

Materials & experimental systems

n/a	Involved in the study	
☐	☒	Antibodies
☒	☐	Eukaryotic cell lines
☒	☐	Palaeontology and archaeology
☒	☐	Animals and other organisms
☒	☐	Human research participants
☒	☐	Clinical data
☒	☐	Dual use research of concern

Methods

n/a	Involved in the study	
☒	☐	ChIP-seq
☒	☐	Flow cytometry
☒	☐	MRI-based neuroimaging

Antibodies

Antibodies used	i) Anti-Cytokeratin 8 mouse monoclonal antibody (C51); Cat # sc-8020, Lot # E1617, Santa Cruz Biotechnology. ii) β-Actin (C4) mouse monoclonal antibody . Cat # sc-47778. Lot # J1119. Santa Cruz Biotechnology. iii) Horseradish peroxidase (HRP)-conjugated goat anti-mouse IgG. Cat # ab205719. Lot # GR3405228-3
Validation	i) Anti-Cytokeratin 8 mouse monoclonal antibody (C51); species reactivity: mouse, rat, human. Validation: Kim et al. JNK/SAPK mediates doxorubicin-induced differentiation and apoptosis in MCF-7 breast cancer cells. Breast Cancer Res. Treat. (2003). Application: Western blotting, immunoprecipitation, immunohistochemistry, solid phase ELISA. ii) β-Actin (C4) mouse monoclonal antibody; species reactivity: mouse, rat, human, avian, bovine, canine, porcine, rabbit, Dictyostelium discoideum and Physarum polycephalum. Validation: Zou et al. MAP4K4 induces early blood-brain barrier damage in a murine subarachnoid hemorrhage model. Neural. Regen. Res. (2021). Application: Western blotting, immunoprecipitation, immunohistochemistry, solid phase ELISA.
