# Supplementary figures and images for: Identifying amyloid-related diseases by mapping mutations in low-complexity protein domains to pathologies
Source: Nat Struct Mol Biol. 2022 May 30;29(6):529–36. doi: 10.1038/s41594-022-00774-y (PMC9205782; doi:10.1038/s41594-022-00774-y)

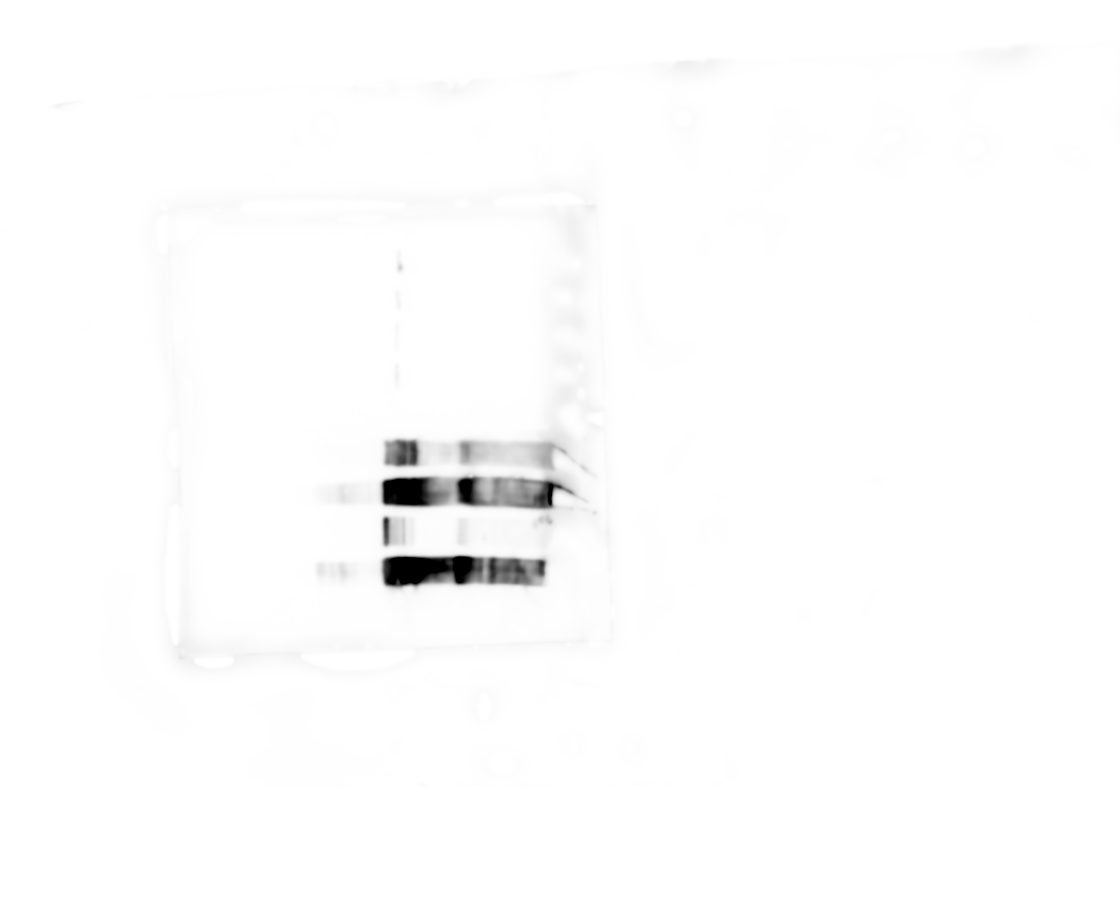

Supplement: Source Data Extended Data Fig. 6 — Unprocessed Western Blot [file 41594_2022_774_MOESM10_ESM.tif]
